# Stag Parties Linger: Continued Gender Bias in a Female-Rich Scientific Discipline

**DOI:** 10.1371/journal.pone.0049682

**Published:** 2012-11-21

**Authors:** Lynne A. Isbell, Truman P. Young, Alexander H. Harcourt

**Affiliations:** 1 Department of Anthropology and Animal Behavior Graduate Group, University of California Davis, Davis, California, United States of America; 2 Department of Plant Sciences and Graduate Group in Ecology, University of California Davis, Davis, California, United States of America; 3 Department of Anthropology and Graduate Group in Ecology, University of California Davis, Davis, California, United States of America; The University of Texas at San Antonio, United States of America

## Abstract

Discussions about the underrepresentation of women in science are challenged by uncertainty over the relative effects of the lack of assertiveness by women and the lack of recognition of them by male colleagues because the two are often indistinguishable. They can be distinguished at professional meetings, however, by comparing symposia, which are largely by invitation, and posters and other talks, which are largely participant-initiated. Analysis of 21 annual meetings of the *American Association of Physical Anthropologists* reveals that within the subfield of primatology, women give more posters than talks, whereas men give more talks than posters. But most strikingly, among symposia the proportion of female participants differs dramatically by the gender of the organizer. Male-organized symposia have half the number of female first authors (29%) that symposia organized by women (64%) or by both men and women (58%) have, and half that of female participation in talks and posters (65%). We found a similar gender bias from men in symposia from the past 12 annual meetings of the American Society of Primatologists. The bias is surprising given that women are the numerical majority in primatology and have achieved substantial peer recognition in this discipline.

## Introduction

Since the 1970s, the percentage of women has increased in scientific societies such as the American Society of Mammalogists, American Psychological Association, American Society of Naturalists, American Society of Primatologists, and American Association of Physical Anthropologists (AAPA) [1], [2]. Indeed, within the AAPA, since the 1970s the field of primatology has had more women than men [3]. However, despite increasing participation by women in science, concerns exist over their retention/advancement, and of their willingness to assert themselves professionally [3]–[6]. One venue where the latter can be examined is at professional meetings.

At annual meetings of the American Society of Mammalogists, participation by women began to increase dramatically shortly after 1979 when posters were introduced as a presentation option [1]. Moreover, the percentage of women as first authors of posters was consistently higher than their percentage for all presentations [1]. However, posters are often viewed as less prestigious than talks [7], [8], and therefore women might be under-selling their research by giving posters instead of talks.

In addition to talks and posters, scientific meetings commonly include symposia. These can be either talks or posters but in all cases, presenters are invited by individuals who organize the symposia around special topics. Presenters are usually scientists who are recognized as having achieved some level of authority on the topic. Thus, symposia are commonly viewed as having even greater prestige than talks.

The perceived differential value of poster, oral, and symposium presentations provides an opportunity to examine whether there are gender differences in self-promotion and other-recognition of influence. Here we examine the contributions of men and women as presenters of research findings within the field of primatology. If any discipline should have women at least equally represented with men in presentations, it would be primatology, a field with a modern reputation as an equal opportunity science [2], [9], [10].

## Methods

We focus here on primatology because it is the first and third authors’ own field of research and there is a strong skew toward women in this field that goes back many years. We examined each of 21 annual meeting issues of the *American Journal of Physical Anthropology* spanning 1992–2012 (all the years in the personal library of LAI) for all presentations on primate behavior/ecology as judged by the session name under which they were included and the title of the presentation. We included all titles in sessions devoted to primate behavior/ecology (e.g., foraging, diet, reproduction, hormones, life history, social organization, development) but not titles in sessions devoted to primate skeletal biology/evolution because these are considered within primate biology and evolution, fields that have not had strong female representation [3]. We also excluded titles in which non-human primates were used only as models to address hominin behavior or evolution because these are typically considered within the category of human evolution, another field in which women have long been in the minority. For symposia focusing on primates, we included all titles even if the presentation was ultimately about hominin behavior/evolution because we were interested more in the behavior of the organizers than the relevance of the topics to primate behavior or ecology.

For each poster, podium, and symposium title, we recorded the gender of the first author based on the first name. We assumed that the first author was also the presenter (unlike in lab sciences, in primatology the senior scientist is not always last). In some cases, the first author’s gender was unclear because 1) first names were designated only with the first initial and we were not familiar with them as individuals, 2) their first names are common among both men and women, or 3) they have international names with which we were unfamiliar. To reduce the number of unclear cases, we used the Internet-based ISI Web of Science to search for other same-authored papers that provided first names, and Google to locate images of them or to identify their first names as for males or females (queried as “Is (name) a boy’s name?” or “Is (name) a girl’s name?”). A few individuals (<3%) were still not identified to gender after searching for them, and these were excluded from analyses dealing with gender. Symposium organizers were similarly gender-identified.

We used the same approach to identify the gender of all symposium participants and organizers in the last 12 annual meeting issues (2000–2011) of the *American Journal of Primatology*, the journal of the American Society of Primatologists. We included all symposia except those specified for student presenters and research grant winners.

We used the VassarStats statistical computational website (http://vassarstats.net/) to conduct statistical tests, all of which were two-tailed non-parametric tests with α set at 0.05.

## Results and Discussion

**Figure 1:**
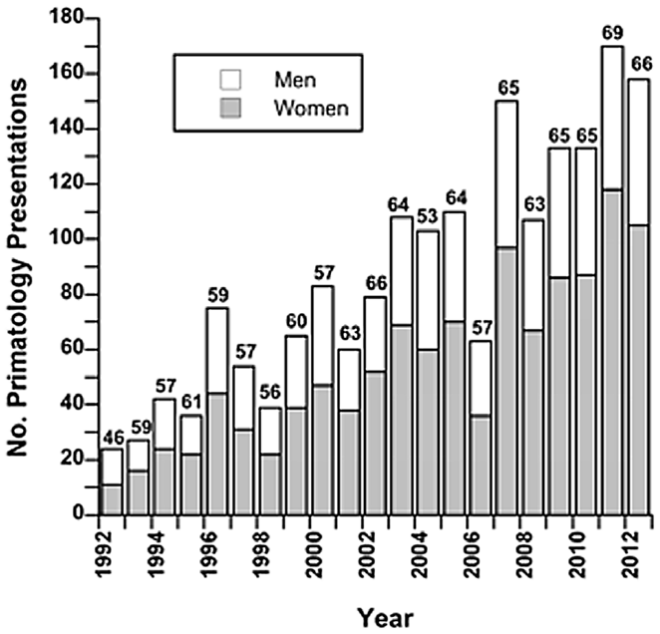
Numbers of presentations in primatology by men and women as first authors at AAPA meetings. Values come from annual meeting issues of the American Association of Physical Anthropologists from 1992–2012. Values above bars are percentages of presentations by women as first authors.

We identified 1874 poster, oral (non-symposium), and symposium presentations in primatology at AAPA meetings between 1992 and 2012, inclusive (range: 28–172 per year). Of these, 1819 were gender-identifiable. Both male and female primatologists have increased their participation at the AAPA meetings over time, although female participation has increased more (Figure 1). In 1992, women contributed 45.8% of presentations in primatology (posters, talks, and symposia combined); by 2012, they contributed 66.5% of all primatology presentations.

Women gave significantly more poster than oral presentations (599 posters vs. 414 talks; χ^2^ = 6.81, p<0.009, df = 1) whereas men gave significantly more talks than posters (283 talks vs. 253 posters; χ^2^ = 12.87, p = 0.0003, df = 1) compared to expected based on their total percentage of participation in posters and talks over the past 21 years. As was the case for the American Society of Mammalogists, the percentage of women as presenters of posters was almost always higher than their percentage for all presentations (including symposium presentations) (Wilcoxon signed-ranks test W = −171, z = 2.96, n = 21, p = 0.003). The exceptions were the years 1992–1994.

One possibility for the bias in favor of women giving posters is that it happens not at the self-selection stage but by others during the development of meeting programs. However, data on presenter requests and decisions from the program committee for the last two years show that men were 12% more likely than women to request talks over posters, although this difference is not statistically significant with only two years of data (men: 45 requests for talks out of 77 presentations; women: 85 requests for talks out of 163 presentations; χ^2^ = 0.6, p = 0.44, df = 1). In addition, men’s and women’s requests to present talks were denied at a statistically similar rate (men: 10 of 45; women: 25 of 85; χ^2^ = 0.22, p = 0.64, df = 1). Nonetheless, non-significant trends in assigning posters to those who preferred to give talks (22% for men vs 29% for women) suggest at least the possibility of some additional bias against women occurring at the selection stage. This possibility should be considered in greater detail in light of a recent study that showed that a bias exists against undergraduate women in science at some American research universities and that it is propagated by both male and female faculty members [11].

A potential problem with posters is that their presenters are often perceived as being less dedicated to research [7], [8]. Posters are also often presented by entry-level researchers, who have a greater chance of leaving science than established researchers. Indeed, a higher percentage of poster presenters (3.8%) than podium (2.5%) and symposium (1.1%) presenters at AAPA meetings could not be gender-identified because they left no history of further scientific accomplishment (χ^2^ = 6.03, p = 0.05, df = 2). Although the gender difference in poster presentations might be posited to have occurred because posters have increased over time and beginning primatologists have not had time to become known to us or to establish a publication history, we found the opposite trend. Of the 55 gender-unidentified presentations, a greater percentage occurred in the first seven years of meetings analyzed (51%; n = 28) than in the central seven years (29%; n = 16) and last seven years analyzed (20%; n = 11). This difference was likely an artifact of the listing of authors using first name initials in earlier years and full first names in later years. It does not differentially affect presentation types.

Discussions about the underrepresentation of women in science include debate about the relative roles of the lack of assertiveness of women and the lack of recognition of them by male colleagues [12]. The two are often indistinguishable. However, at meetings the two can be distinguished by comparing symposia, which are largely by invitation, and posters and other talks, which are largely participant-initiated and, importantly, viewed as less prestigious than symposia.

**Figure 2:**
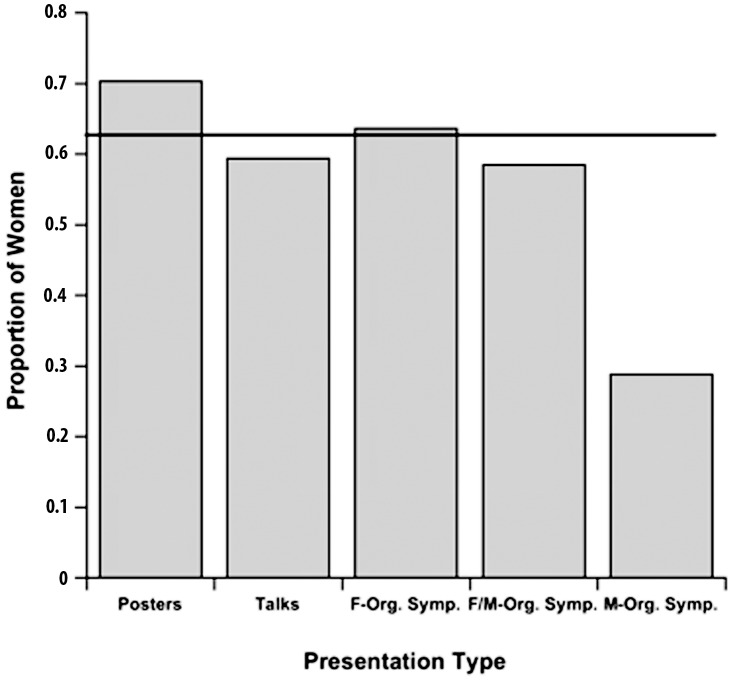
Proportion of women as first authors of posters, talks, and symposia at AAPA meetings. The average proportion for all presentations with women as first authors over a 21-year period of annual meetings of the American Association of Physical Anthropologists is indicated by the solid black line. F-Org. Symp.: symposia organized by women only; F/M Org. Symp.: symposia organized jointly by women and men; M-Org. Symp.: symposia organized by men only.

First, whereas women were first authors on 65.4% (1013 of 1549) of podium and poster sessions they were first authors on only 47.4% (128 of 270) of symposium presentations (χ^2^ = 31.06, p<0.0001, df = 1). Second, participation by men and women in symposia differed strongly with the gender of the organizers (χ^2^ = 29.43, p<0.0001, df = 2). In female-organized symposia, 63.6% of presenters were women (63 of 99), not significantly different from the percentage (65.4%) of women presenting talks and posters (χ^2^ = 0.06, p = 0.81, df = 1). In symposia organized by both sexes, women constituted 58.5% of first authors (31 of 53), also not significantly different from their percentage as first authors in talks and posters (χ^2^ = 0.79, p = 0.37, df = 1). However, in male-organized symposia, only 28.8% of first authors were women (34 of 118), less than half the percentage of women participating in talks and posters (χ^2^ = 61.26, p<0.0001, df = 1) (Figure 2).

In case this bias was a peculiarity of anthropologically based primatology, we also examined male- and female-organized symposia from the past 12 years (2000–2011) of annual meetings of the American Society of Primatologists, whose members come from more diverse academic backgrounds [13]. We found the same pattern. In female-organized symposia, 58.1% (104 of 179) of first authors were women, but in male-organized symposia, only 38.8% (31 of 80) were women (χ^2^ = 7.54, p<0.006, df = 1). In 2002, women comprised 57.7% of the membership of this organization [13].

Participants of symposia are invited by organizers. They are typically invited because they are already established in their careers by organizers also already established. Nevertheless, cohort, or career-stage, effects to explain the obvious gender bias of male organizers can be ruled out. If there were career-stage effects, lower participation by women in male-organized symposia should have also been seen in symposia organized by women. As shown above, however, no gender bias in invitees exists when a woman is an organizer. A career-stage effect might also be suspected if there had been an increase in the percentage of women in symposia over time. This was not the case, however. Over successive seven-year increments, women constituted an average of 22% (1992–1998), 24% (1999–2005), and 28% (2006–2012) of the participants in male-organized symposia, a slight increase but still well below the values for symposia organized by women over the same time periods: 80%, 50%, and 72%. Women have been a part of primatology for so long [2], [9] that any career-stage effect that might have existed likely disappeared before the years of this study. Indeed, there are now so many women in late career stage that more women are being recognized for their lifetime contributions to primatology. Since 2006, five of seven scientists awarded the American Society of Primatologists’ (ASP) Distinguished Primatologist Award for outstanding careers in primatology have been women. Several of these are also frequent participants at AAPA meetings. Similarly, since 1992, six of 11 ASP presidents and presidents-elect have been women, an achievement possible only for those at a mature career stage.

Another possibility for the gender bias less easy to rule out is that men are more homophilic than women [14]. Homophily is preferential interaction with others who have similar attitudes, beliefs, or personal characteristics [14], [15]. One benefit of homophily appears to be easier communication and increased behavioral predictability, both of which may increase productivity [16]. Unlike podium and poster sessions, symposium sessions require the organizer(s) to work with multiple presenters in the months leading to the symposia and often beyond if the presentations are published in an edited volume. Inviting those of the same gender or those with whom one already has a relationship may improve the organizer’s efficiency in the task. If this is the case, why are only male primatologists homophilic? The short answer may be because they can be.

Women may be less homophilic than men in practice because men are still highly influential in academic departments and women tend to gain greater professional success when they have instrumental relationships with or sponsorships from men [2], [13], [17]. Indeed, when the influence or power differential between men and women is removed, women may be as homophilic as men: one study reported that 78% of first authors working on male primates only were men and 77% of first authors working on female primates only were women [18].

Regardless of whether the cause of the gender bias against women in invitations to prestigious symposia is due to homophily or another cause, its discovery requires attention in a field that is exemplary in being gender-blind in so many other ways [2].

A recent study suggested that women in primatology face a “glass ceiling” in promotions to high-level academic positions, e.g., full professors [4]. Several explanations for this were suggested, including a tendency for women to drop out of academic life or to rise through the ranks of the academy more slowly in response to familial considerations and a bias toward men among high-level academicians, the majority of whom are also men. The case has been made for these explanations in other sciences as well [6]. We suggest, however, that if a glass ceiling exists in primatology, it is not often constructed by primatologists. Even if women are the majority in primatology, primatology is only a small part of almost all relevant academic departments. In contrast to the obvious and easily correctable bias against women in male-organized symposia at AAPA and ASP meetings, the glass ceiling may be something largely out of the control of primatologists because decisions on hiring and promotion are often made at the departmental level or higher [19], [20].

## References

[1] Genoways HH, Freeman PW (2001) Evolution of a scientific meeting: eighty annual meetings of the American Society of Mammalogists, 1919–2000. J Mammal 82: 582–603.

[2] Fedigan LM (1994) Science and the successful female: why there are so many women primatologists. Am Anthropol 86: 529–540.

[3] Turner TR (2002) Changes in biological anthropology: results of the 1998 American Association of Physical Anthropology membership survey. Am J Phys Anthropol 118: 111–116. 12012363 10.1002/ajpa.10062

[4] Addessi E, Borgi M, Palagi E (2012) Is primatology an equal-opportunity discipline? PLoS ONE 7: e30458 doi:10.1371/journal.pone.0030458. 22272353 10.1371/journal.pone.0030458 PMC3260283

[5] Ley TJ, Hamilton BH (2008) The gender gap in NIH grant applications. Science 322: 1472–1474. 19056961 10.1126/science.1165878

[6] Williams WM, Ceci SJ (2012) When scientists choose motherhood. Am Scient 100: 138–145. 10.1511/2012.95.138 PMC3939045 24596430

[7] Macintosh-Murray A (2007) Poster presentations as a genre in knowledge communication: a case study of forms, norms, and values. Science Commun 28: 347–376.

[8] Fuentes A (2003) Fear not the poster. Anthropol News 44: 14.

[9] Fedigan LM (1997) Is primatology a female science? In: Hager L, ed. Women in Human Evolution. London: Routledge, 56–75.

[10] Schiebinger L (2000) Has feminism changed science? Signs 25: 1171–1175. 17089478 10.1086/495540

[11] Moss-Racusin CA, Dovidio JF, Brescoli VL, Graham MJ, Handelsman J (2012) Science faculty's subtle gender biases favor male students. Proc Natl Acad Sci USA 109: 16474–16479. 22988126 10.1073/pnas.1211286109 PMC3478626

[12] Hrdy SB (1986) Empathy, polyandry, and the myth of the coy female. In: Bleier R, ed. Feminist Approaches to Science. New York: Pergamon. 119–146.

[13] Schapiro SJ (2003) Member characteristics of the American Society of Primatologists through 2002. Am J Primatol 61: 45–52.

[14] Ibarra H (1992) Homophily and differential returns: sex differences in network structure and access in an advertising firm. Admin Sci Quart 37: 422–447.

[15] Lazarsfeld PF, Merton RK (1954) Friendship as a social process: a substantive and methodological analysis. In: Berger M, Abel T, Page CH, eds. Freedom and control in modern society. New York: Van Nostrand. 18–66.

[16] Lincoln JR, Miller J (1979) Work and friendship ties in organizations: a comparative analysis of relational networks. Admin Sci Quart 24: 181–199.

[17] Hewlett SA, Peraino K, Sherbin L, Sumberg K (2010) The sponsor effect: breaking through the last glass ceiling. Harvard Bus Rev Res Rep 1–85.

[18] Holmes DJ, Hitchcock CL (1997) A feeling for the organism: An empirical look at gender and research choices of animal behaviorists. In: Gowaty PA, ed. Feminism and evolutionary biology: boundaries, intersections and frontiers. New York: Chapman and Hall. 184–202.

[19] Monroe K, Ozyurt S, Wrigley T, Alexander A (2008) Gender inequality in academia: bad news from the trenches, and some possible solutions. Perspect Politics 6: 215–233.

[20] Bird SR (2011) Unsettling universities’ incongruous, gendered bureaucratic structures: a case-study approach. Gender Work Org 18: 202–230.

